# A review of clinical profile, complications and antibiotic susceptibility pattern of extensively drug-resistant (XDR) Salmonella Typhi isolates in children in Karachi

**DOI:** 10.1186/s12879-021-06599-2

**Published:** 2021-09-03

**Authors:** Saba Shahid, Marvi Mahesar, Nida Ghouri, Saba Noreen

**Affiliations:** 1 Department of Pediatrics, The Indus Hospital, Karachi, Pakistan; 2 Indus Hospital Research Centre, The Indus Hospital, Karachi, Pakistan

**Keywords:** XDR S.Typhi, Seasonality, Children, Antibiotics

## Abstract

**Background:**

Enteric fever is a systemic infection caused by *Salmonella* *enterica serovar Typhi* and *Salmonella* *enterica serovar Paratyphi A*, *B*, and *C*. There is an emergence of Typhoid fever caused by extensively drug-resistant Salmonella Typhi strain called XDR S.Typhi. This strain is resistant to recommended first-line antibiotics and cephalosporins. WHO estimated 5274 cases of XDR S.Typhi in Karachi from November 2016 to December 2019. This study aims to determine clinical course, complications and response to treatment of XDR S.Typhi among the pediatric population coming to Indus Hospital.

**Method:**

We reviewed the records of children who had culture-proven XDR S.Typhi infection at Indus Hospital from July 2017 to December 2018. A pre-designed data abstraction form was used to record information about seasonality, demographic details, clinical features and course, treatment, complications and outcomes of the cases of XDR S.Typhi.

**Results:**

The records of 680 children were reviewed**.** The median (IQR) age of the patients was 5 (2–8) years. More than half (n = 391, 57.5%) of the patients were males. The outcomes were recorded in 270 (40%) patients. Out of these, 234 (86.7%) children got cured within 14 days, while a delayed response to antibiotics was noted in 32 (11.9%) children. Seventy-six (29%) children recovered on a combination of meropenem and azithromycin, 72 (27%) got cured on azithromycin alone, while 15 (6%) responded to meropenem alone.

**Conclusion:**

Our review indicated that children under 5 years of age were affected more with XDR S.Typhi. Azithromycin alone or in combination with meropenem were effective antibiotics for treating XDR S.Typhi in children.

## Background

Enteric fever is a systemic infection caused by Gram-negative bacteria, *Salmonella enterica serovar Typhi*, *Salmonella* e*nterica serovar Paratyphi A*, *B*, and *C* [1]. The disease is estimated to affect approximately 11–21 million individuals globally on an annual basis and has a high mortality rate [2]. Recent data showed that globally 200,000 deaths result annually due to enteric fever [3]. The burden of disease of enteric fever is the highest in Asia; 93% of the global cases are reported from within this region [3]. Southeast Asia has the third-highest incidence within the region, with approximately 110 cases/100,000 population. The estimated incidence of enteric fever in Pakistan was 413/100,000 in children aged 2–4 years and 573/100,000 in children aged 5–15 years [1].

Over time, Salmonella Typhi has developed resistance to many antibiotics, resulting in the emergence of multi-drug resistant Salmonella Typhi (MDR S.Typhi). These strains have shown resistance to first-line drugs, namely ampicillin, chloramphenicol and trimethoprim-sulfamethoxazole. However, they are sensitive to third-generation cephalosporins [4]. This strain of enteric fever has been endemic in countries like Pakistan, India, Nepal and Bangladesh since the 1980s [4]. However, a review of S.typhi and Paratyphi-A conducted in Pakistan from 2009 till 2011 showed an increased frequency of MDR S.Typhi (91.7%) and two cases of S.Typhi resistant to cephalosporin [5].

In November 2016, a massive outbreak of ceftriaxone-resistant S.Typhi was reported among children residing in Hyderabad, Pakistan. Around 486 cases were reported, and consumption of contaminated drinking water was linked with the infection [5]. These strains were called extensively drug-resistant Salmonella Typhi (XDR S.Typhi) as they showed resistance to all the recommended antibiotics for typhoid fever, including third-generation cephalosporin. In addition, the strains of XDR S.Typhi showed sensitivity to either carbapenem (meropenem and ertapenem) or azithromycin [6]. Since the outbreak of typhoid fever caused by XDR S.Typhi in Hyderabad, many other similar cases have been reported. WHO estimated 5274 cases of XDR S.Typhi in Karachi from November 2016 to December 2019 [7]. In Pakistan, cases of XDR S.Typhi are routinely managed with azithromycin and meropenem alone or in combination.

XDR S.Typhi is a new strain and may have unique clinical manifestations and outcomes compared to MDR S.typhi infection. Unfortunately, there is a scarcity of data on clinical features and response to treatment in children suffering from XDR S.Typhi nationally and in other parts of the world. Therefore this study is done to determine clinical course, complications and outcomes of XDR S.Typhi among the pediatric population coming to Indus Hospital. Primary endpoints included clinical manifestations, complications, response to treatment and outcome of the participants. Secondary endpoints included the seasonality of the infection and geographic distribution of the cases.

## Methods

### Study design and data collection

A retrospective chart review of blood culture-confirmed XDR S.Typhi cases was conducted at The Indus Hospital, Korangi campus from 1st July 2017 to 31st December 2018. The Indus Hospital is a free, multi-disciplinary tertiary care hospital situated in Karachi. Pediatric services include 115 beds. Pediatric services at Korangi campus include pediatric oncology, critical care and general ward.

Children with ages ranging from birth till 15 years were included in the study. Data from both inpatient and outpatient (OPD) records were obtained. Blood culture-confirmed cases of XDR S.Typhi, which showed resistance to the five classes of antibiotics (ampicillin, chloramphenicol, trimethoprim-sulfamethoxazole, fluoroquinolones, and 3rd generation cephalosporin (ceftriaxone or cefixime), were included in the study. All children with XDR S.Typhi identified during the study period were included. Clinical features and lab investigations done at the time of presentation were noted for those children who left against medical advice (LAMA) or did not seek treatment or returned to hospital for follow-up visits. Outcomes were noted for those children who had a complete record. After discharge, admitted children were followed in OPD on day 14 to check the clinical response to treatment. The children were considered cured if fever defervescence was achieved within 14 days of starting antibiotics. For children who did not show fever defervescence on day 14, antibiotics were continued, and blood culture was repeated. These children were considered to have delayed response to antibiotics. The second follow-up was done for late responders on day 21. During the second follow up clinical response to antibiotics was noted, and blood culture was repeated if fever persisted. Children who missed their appointment were reminded through phone calls as a routine practice in the institute.

A pre-designed data abstraction form was used to record detailed information about the month-wise distribution of cases, patient demographics, signs and symptoms, clinical course, time in days to defervescence from infection, details of antimicrobial therapy, complications and outcomes. In addition, laboratory parameters were also recorded. These included complete blood count (CBC), C-reactive protein (CRP), liver function test (LFTs), serum electrolytes, creatinine, cerebrospinal fluid analysis, ultrasound and CT scan reports. Data of complications retrieved from the medical record included electrolyte imbalances, haematological complications like cytopenias, cholecystitis, hepatitis, ascites, pleural effusion, shock, renal dysfunction, neurological complications like fits, encephalitis, encephalopathy and aphasia.

Treatment options noted in our study reflected the treatment protocols of our institute. All children suspected of having enteric fever were empirically treated with cefixime or ceftriaxone, depending on disease severity. Upon confirmation of XDR S.Typhi based on blood culture, antibiotics were switched to either azithromycin or meropenem. Uncomplicated cases of XDR S.Typhi were given azithromycin for 10 days, while complicated cases were treated with meropenem for 14 days. Admitted children receiving meropenem were discharged on oral azithromycin to complete treatment of 14 days. Meropenem was given Intravenously (IV) at a dose of 20–40 mg/kg three times a day, while azithromycin was given orally at 20 mg/kg/day.

Three to five ml of venous blood sample was drawn and blood was inoculated into BacT/Alert culture bottles of BD company. Additional subcultures were performed if bacterial growth was suspected on visual identification. Subculture was done on Mac-Conkey agar and Blood agar plates. Non-lactose fermenting colonies isolated on Mac-Conkey agar were biochemically tested for Salmonella typhi and confirmed by Salmonella specific anti-sera. Biochemical tests done to identify Salmonella species included motility, indole, citrate utilization, triple-sugar iron and sulfide production test.

XDR S.Typhi was identified on basis of antibiotic sensitivity which was tested by Kirby–Bauer disc diffusion technique on Mueller Hinton agar with standard antimicrobial discs. Antibiotic susceptibility for ampicillin, chloramphenicol, trimethoprim-sulfamethoxazole, ciprofloxacin, cefixime, ceftriaxone, imipenem, ertapenem, meropenem and azithromycin was performed according to CLSI guidelines 2018 [8]. For quality control *E. coli* ATCC 25922 was used as a reference strain in the disk diffusion susceptibility test.

We obtained approval from the ethical review committee (ERC) of our institute. We were granted ERC exemption, as this study is a retrospective chart review.

### Case definitions

Non-resistant typhoid fever: Typhoid fever caused by S.Typhi or S.Paratyphi A, B or C strains sensitive to first-line drugs (ampicillin, chloramphenicol and trimethoprim-sulfamethoxazole, quinolone) and third-generation cephalosporins (cefixime and ceftriaxone) [5].
Multi-drug resistant Salmonella Typhi (MDR S-Typhi): Typhoid fever caused by S.typhi and or S.paratyphi A, B or C strains which are resistant to first-line drugs (ampicillin, chloramphenicol, and trimethoprim-sulfamethoxazole) [5].
Extensively drug-resistant Salmonella Typhi (XDR S.Typhi): Typhoid fever caused by *S.typhi* strains that are resistant to all the recommended antibiotics for typhoid fever, including ampicillin, chloramphenicol, trimethoprim-sulfamethoxazole, quinolones (nalidixic acid/ciprofloxacin) and third-generation cephalosporin( cefixime and ceftriaxone) [5].
Cured: Cured was defined as defervescence of fever within 14 days of starting antibiotics.
Delayed response: It was defined as either persistence of fever or positive blood culture beyond 14 days of treatment.
Treatment failure: It was defined as persistence of fever or positive blood culture beyond 21 days of starting antibiotics or relapse of disease within 30 days of completion of antibiotics.
Lost to follow up: It was defined as children who did not seek treatment or returned to the hospital for a follow-up visit after the initial visit.
Renal impairment: Increased creatinine > 1.5 times upper limit of normal or decreased in urine output < 0.5 ml/kg/h for 6 h [9].
Hepatitis: Deranged liver function tests with ALT more than twice the reference value with or without hyperbilirubinemia, impaired coagulation and hypoglycemia.
Cholecystitis: Evidence of inflamed gall bladder sludge in gall bladder on ultrasound.
Haematological complications: A decrease in two cell counts from given lab references were considered bi-cytopenia. A decrease in three cell counts from given lab references were considered pancytopenia.
Encephalopathy: Changes in mental status, confusion or stupor with normal CSF findings.
Encephalitis: Changes in mental status, confusion or stupor or signs of meningeal irritation with abnormal CSF findings.
Diarrhoea: Presence of loose or watery stools three times or more per day [10].

### Statistical analysis

Data were analysed using SPSS version 24. Descriptive statistics were run for all continuous variables, exploring skewness and kurtosis. Mean with standard deviation was reported for normal distribution, while median with interquartile range was reported for skewed distribution.

## Results

A total of 1518 patients had blood culture positive for enteric fever during the study period. Out of these, 1341 patients were children, and 177 patients were adults.

**Fig. 1:**
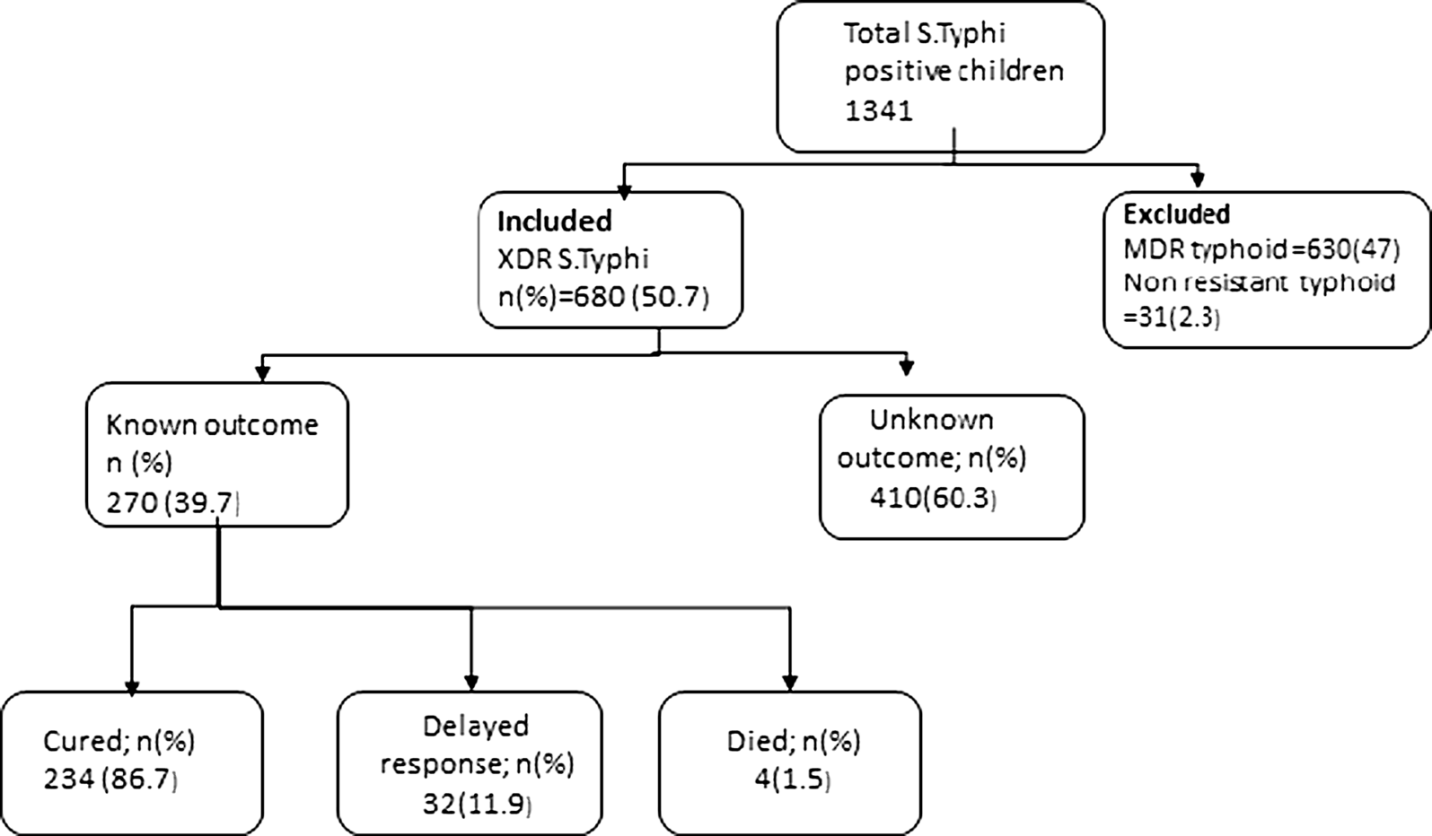
Flow chart of patient inclusion, exclusion and outcomes

Out of 1341 pediatric patients, 661 children were excluded from the final analysis as they had MDR typhoid or non-resistant typhoid infection. However, XDR S.Typhi infection was present in 680 children, and they were included in the final analysis (Fig. 1). More than half (n = 391, 58%) of the patients were males. The patients’ median (IQR) age was 5 (IQR 2–8) years, with minimum and maximum 0–14 years. Out of these patients, 612 (90%) children presented to the emergency department, followed by OPD 65(9.6%). Around 101 (15%) patients were admitted to the hospital, 98 (97%) patients were admitted to the general ward, and the rest were admitted to the intensive care unit for treatment (Table 1).

**Table 1:** Demographic characteristics, clinical features, investigations, complications and outcome of study participants

Demographic features	Number (%)
Male	391 (58)
Female	289 (43)
Age in years median (IQR)	5 (2–8)
Place of presentation	
E/R	612 (90)
OPD	65 (9.6)
Inpatient	3 (0.4)

Clinical features on presentation (680)	Number (%)
Fever	680 (100)
Vomitting	242 (36)
Diarrhea	174 (26)
Anorexia	140 (21)
Cough	126 (19)
Abdominal pain	125 (18)
Hepatomegaly	63 (9)
Splenomegaly	22 (3)
Urinary symptoms	14 (2)
Rash	12 (2)
Bleeding per rectum	4 (1)

Lab investigations	Number (%)
Raised CRP > 5 (415)	395 (95)
Hyponatremia (severe + moderate combined) (178)	74 (41)
Acidosis (178)	65 (37)
Hypokalemia (severe + moderate combined) (178)	59 (33)
Severe thrombocytopenia (< 50,000 × 10^9^/L) (676)	24 (4)
Raised serum creatinine (151)	4 (3)
Severe anemia (Hb < 5 gm/dl) (676)	9 (1)

Complications	Number (%)
Mesentric lymphadenopathy (on abdominal ultrasound), (62)	11 (18)
Encephalopathy (101)	16 (16)
Hepatitis, LFT (81)	11 (14)
Peritoneal free fluid (on abdominal ultrasound) (62)	8 (13)
Bi-cytopenia (676)	67 (10)
Pleural effusion (on abdominal ultrasound) (62)	6 (10)
Cholecystitis (on abdominal ultrasound) (62)	4 (6)
Shock (seen in 101 admitted patients)	5 (5)
Encephalitis (seen in 101 admitted patients)	4 (4)
Aphasia (seen in 101 admitted patients)	4 (4)
Pancytopenia, done in 676	16 (2)

Outcomes	
Patients with known outcomes (270)	
Cured	234 (87)
Delayed response	32 (12)
Died	4 (1)
Patients with unknown outcomes (410)	
Lost to follow up	351 (86)
LAMA/referred out	59 (14)

*LAMA* leave against medical advice

**Fig. 2:**
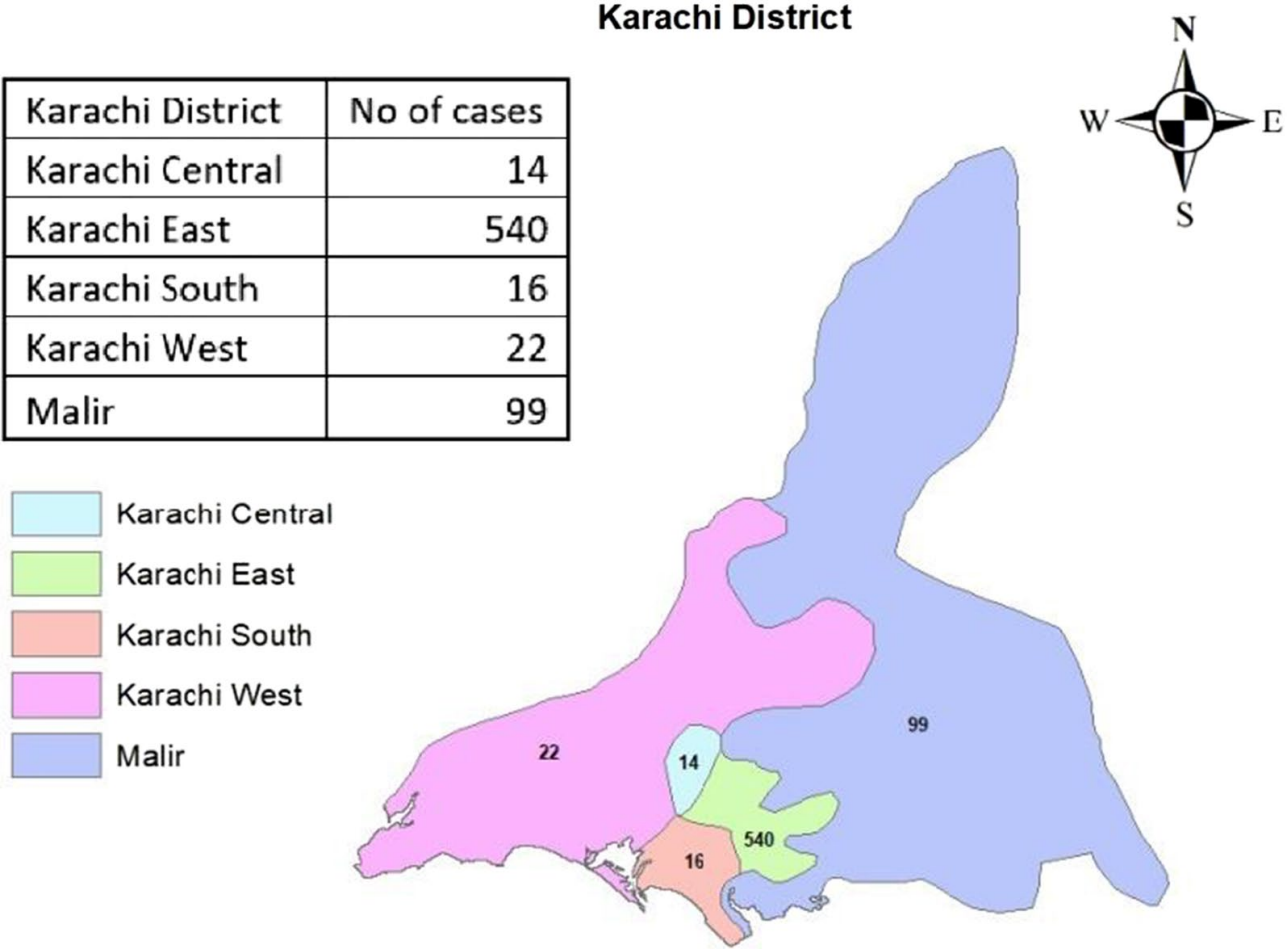
Geospatial map of XDR Enteric cases in Karachi

**Fig. 3:**
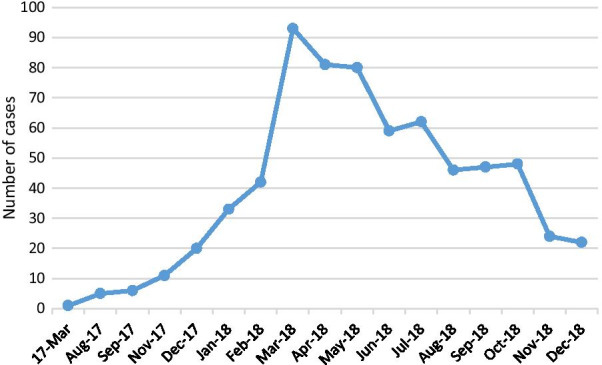
Monthly distribution of XDR S.Typhi cases

The majority of children, 540 (79%), came from district East (Fig. 2). Two seasonal peaks were identified in the year 2018. One was in February-May 2018, and the second peak was observed in August–October 2018 (Fig. 3).

Out of 101 admitted patients, 69 children were cured within 14 days, while 32 patients had delayed response to treatment, as their blood culture was negative after 14 days. The average hospital stay of admitted children was 6 (IQR 3–9) days. The most frequent complications observed in admitted patients were mesenteric lymphadenopathy (18%), encephalopathy (16%) and hepatitis (14%) (Table 1). Among the admitted patients, 4 cases of encephalitis were noted, out of which three children had aphasia on presentation. All children with encephalitis had full recovery without any residual weakness or speech difficulty. Mortality was seen in four admitted children. All the deceased children were less than 4 years of age and died within 3 days of admission except for one child who died after 14 days of admission. Among the children who died, two received meropenem, one child was given a combination of meropenem and azithromycin, and one child received a combination of ceftriaxone and meropenem (Table 2). A neonate was identified among the deceased children who acquired XDR S.Typhi through vertical transmission. Causes of death included multi-organ dysfunction, pulmonary haemorrhage, renal impairment and electrolyte imbalances.

**Table 2:** Clinical outcomes of patients on different antimicrobial therapy

Antimicrobial group n (%)	Cured (266) n (%)		Died (4) n (%)	Unknown outcomes (410) n (%)
	Response within 14 days (234)	Response after 14 days (32)		
Azithromycin only	69 (29.5)	3 (9.4)	–	41 (10)
Combination of Azithromycin and Meropenem	63 (26.9)	13 (40.6)	1 (25)	5 (1.2)
Meropenem only	12 (5.1)	3 (9.4)	2 (50)	10 (2.4)
Cefixime	3 (1.3)	–	–	26 (6.3)
Ceftriaxone	3 (1.3)	1 (3.1)	–	74 (18)
Ciprofloxacin	1 (0.4)	–	–	
Other combinations*	70 (29.9)	11 (34.4)	1 (25)	36 (8.8)
Not noted	13 (5.6)	1 (3.1)	–	218 (53.2)

*Other combinations included ceftriaxone and meropenem, ceftriaxone and azithromycin, imipenem and azithromycin

Outcomes were recorded in 270 (40%) patients; (Table 1). Out of these, 234 (86.7%) children got cured, 196 children got cured based on fever defervescence, while 38 children had fever defervescence and culture clearance within 14 days. Delayed response to antibiotics beyond 14 days was noted in 32 (11.9%) children (Table 2). However, all children with delayed response got fever defervescence by 21 days of starting antibiotics. Thus, we did not find any treatment failure in our study.

Out of 270 children whose outcomes were known, 76 (28%) recovered on a combination of meropenem and azithromycin, 72 (27%) got cured on azithromycin alone, while 15 (6%) responded to meropenem alone. Eighty-one (30%) children got cured on other combinations, including ceftriaxone, carbapenem, and azithromycin (Table 2). Delayed response beyond 14 days was noted in 32 (12%) children. Out of these, 24 children (75%) were receiving various combinations of antibiotics, while seven (21%) children received single antibiotic. Antimicrobial information was missing for one child (Table 2).

## Discussion

Enteric fever caused by S.typhi continues to pose as a health burden globally, with the incidence being highest in low to middle-income countries (LMIC) due to poor infrastructure of public health [11]. According to the World Health Organization [12], Pakistan faced the largest outbreak of XDR S.Typhi in Hyderabad in November 2016, followed by a similar outbreak in Karachi. XDR S.Typhi is a novel strain that belongs to the H58 lineage. It has plasmid-encoded resistance and extended-spectrum β-lactamase (ESBL) gene responsible for resistance to both first and second-line antibiotics [13]. Klemm et al. investigated the molecular epidemiology of XDR S.Typhi and found that these strains were also isolated from four different countries besides Pakistan, including India, Bangladesh, United Kingdom and Iraq. Of these, plasmid isolated from Iraqi strains were different while the rest of the strains were similar to Pakistan [13].

Out of 1518 total positive blood cultures for typhoid fever, 1341 (88%) belonged to children. The median age of children with XDR S.Typhi was 5 (IQR 2–8) years. Literature from other parts of Pakistan has also reported a higher frequency of infection among children less than 5 years of age [7, 14]. This could be explained by the fact that children have lower immunity and require lower bacterial doses to develop infection [15]. Most of the participants in our study belonged to the East district of Karachi, probably because Indus Hospital lies in the catchment area of this district. Many residential areas located in district East of Karachi comprise peri-urban slums, having unhygienic conditions, inadequate sewerage facilities and consumption of pipe-borne portable water supply by the people. The high burden of disease in these areas could be due to contaminated drinking water and mixing of drinking and sewage water, a finding which was also observed in Hyderabad [6].

Typhoid fever has been associated with considerable seasonal variations in different parts of the world [16]. In Pakistan, MDR typhoid fever peaks have been noted in May–June and in October. Both these periods are associated with monsoon rains in Pakistan. It has been postulated that seasonality is linked to increased consumption of contaminated local drinks and ice cream during the hot season and post-monsoon contamination of drinking water with rainwater [17]. We selected the year 2018 for our study because there was an outbreak of XDR S.Typhi in Karachi during this time. We observed two peaks in the number of cases, one in February–May 2018 and the second in August–October 2018. In addition, we observed a peak number of cases from February to May 2018, which was a dry period in Karachi. This deviation from the usual seasonal pattern of the rainy season could be attributed to the XDR S.Typhi outbreak in many cities of Pakistan simultaneously in the year 2018. This finding is supported by another survey conducted in Lahore by Latif et al., who also reported peak cases of XDR S.Typhi from January to April 2018 [18]. There is a possibility that intercity travelling during these cities had contributed to the spreading of infection.

Electrolyte imbalance was one of the most common reasons for hospital admission and can be attributed to vomiting and diarrhoea seen in our patients. Hyponatremia was seen in 74 (41%) children and hypokalemia in 59 (33%) children. The hypovolemic shock was seen in 5 (5%) children. All children with shock responded to fluids and inotropes except for one child who passed away. Neurological complications, which included encephalopathy and encephalitis, were seen in 20 children. Out of these, 15 children had seizures. Three children with fits had hyponatremia, four had encephalitis, and eight children had encephalopathy. Leung et al. [19] have reported 48 cases of enteric fever with encephalopathy. They found a strong correlation of encephalopathy with dehydration and low white blood cell count (p-value < 0.001). They postulated that heightened immune response in typhoid fever could lead to neurological complications. There is a possibility that children in our cohort also had an immunological mediated neuronal injury.

Four children developed aphasia, out of which three had encephalitis, and one had encephalopathy. All children recovered completely without any deficit. Few cases of aphasia caused by enteric fever have been reported [20, 21]. The literature reports multiple factors like electrolyte imbalance, cerebral injury and neurotoxins-associated injury in Broca's speech as the cause of motor aphasia in Enteric fever [20].

Mortality was seen in four children. Three children passed away within 3 days of admission, while the fourth child, a neonate, died in the 2nd week of infection. Delay in seeking medical treatment could be a contributing cause of early deaths in these children. Three hundred fifty-one children were lost to follow up in our study. The most probable explanation for this could be poverty, as most of our study participants belonged to low socio-economic strata. These patients may find it challenging to afford travelling expenses.

In our study, 76 (29%) children recovered on a combination of meropenem and azithromycin, 72 (27%) got cured on azithromycin alone, while 15 (6%) responded to meropenem alone.

Tayyaba et al. studied antibiotic susceptibility of XDR S.Typhi in Karachi and found equal susceptibility of XDR S.Typhi strains to both azithromycin (95%) and meropenem (97%) [22]. other studies also reported equal cure rates with both the drugs [23, 24]. The number of cured children were almost similar in antimicrobial groups of azithromycin alone and azithromycin-meropenem combination, 76 (28%) vs 72 (27%). This finding has important future implications as our data suggests that XDR S.Typhi can be successfully treated with oral azithromycin, which will reduce the cost of treatment and hospital admission. Qureshi et al. [25] also reported that treatment of XDR S.Typhi by meropenem is more expensive than azithromycin. They reported an average daily cost of treatment for azithromycin to be US$5.87 versus US$88 for meropenem.

We found late responders but no treatment failure in our cohort. There is a possibility that children who had delayed response harboured XDR S.Typhi strains with a higher minimum inhibitory concentration (MIC) to azithromycin and meropenem. Since we did not perform MIC in our institute, we were unable to determine the association of MIC with clinical response.

Seven children got cured on cefixime, ceftriaxone and ciprofloxacin alone, although they were reported as resistant to those antibiotics. One explanation for this phenomenon could be that the resistant strains had higher MIC breakpoints and were missed by our lab due to lack of MIC. Another possibility could be that these patients also received azithromycin and did not communicate this information to us.

The strength of this study is that it is one of the few studies which examines the clinical course, outcomes, response to treatment and complications of XDR strain of S.Typhi amongst the pediatric population within Pakistan. However, the limitations of this study include the lack of use of MIC for culture and sensitivity, which would provide more information about response to antibiotics. Furthermore, more than half the children were lost to follow-up, and we could not determine the reasons for missed appointments. In addition, retrospective data had missing information on socio-demographics like drinking water quality, hygiene practices and the number of households.

## Conclusion

This study confirms that XDR S.Typhi is common in children under 5 years of age. The use of azithromycin alone or in combination with meropenem for the treatment of XDR S.Typhi has shown promising results. However, the effectiveness of these regimens needs to be further investigated through randomised clinical trials. We advocate increasing nationwide awareness about the consumption of safe water, antibiotic stewardship and immunisation practices of children.

## Data Availability

The datasets used and analysed during the current study are available from the corresponding author on reasonable request.

## Abbreviations

XDR: Extensively drug-resistant
MDR: Multi-drug resistance
WHO: World Health Organisation
IQR: Interquartile range
S.typhi: Salmonella Typhi
HMIS: Health Management Informatics System
CBC: Complete blood count
LFT: Liver function test
ALT: Alanine transaminase
CSF: Cerebrospinal fluid
